# Ferroptosis: Biochemistry and Biology in Cancers

**DOI:** 10.3389/fonc.2021.579286

**Published:** 2021-04-01

**Authors:** Zhiyuan Shi, Lei Zhang, Jianzhong Zheng, Huimin Sun, Chen Shao

**Affiliations:** ^1^Department of Urology, Xiang’an Hospital of Xiamen University, Xiamen, China; ^2^School of Public Health, Xiamen Univerisity, Xiamen, China; ^3^Clinical Central Research Core, Xiang’an Hospital of Xiamen University, Xiamen, China

**Keywords:** ferroptosis, cancers, amino acid metabolism, iron metabolism, lipid metabolism

## Abstract

The challenge of eradicating cancer is that cancer cells possess diverse mechanisms to protect themselves from clinical strategies. Recently, ferroptosis has been shown to exhibit appreciable anti-tumor activity that could be harnessed for cancer therapy in the future. Ferroptosis is an iron-dependent form of regulated cell death that is characterized by the oxidization of polyunsaturated fatty acids (PUFAs) and accumulation of lipid peroxides. Ferroptosis has been closely correlated with numerous biological processes, such as amino acid metabolism, glutathione metabolism, iron metabolism, and lipid metabolism, as well as key regulators including GPX4, FSP1, NRF2, and p53. Although ferroptosis could be involved in killing various cancer cells, multiple aspects of this phenomenon remain unresolved. In this review, we summarize the biochemistry and biology of ferroptosis in diverse cancers and discuss the potential mechanisms of ferroptosis, which might pave the way for guiding cancer therapeutics.

## Introduction

Cancer has been widely recognized as the second leading cause of death worldwide (1). According to recent statistics on cancers in 185 countries, there were 19.3 million new cancer cases (18.1 million excluding nonmelanoma skin cancer) and 10 million cancer deaths (9.9 million excluding nonmelanoma skin cancer) worldwide in 2020 (2). Although several therapeutic strategies such as surgery, radiotherapy, chemotherapy, ablation, and organ transplantation have been utilized to fight against cancer, many malignant cancers still do not show satisfactory results after treatment. The two major causes of cancer-related death are metastasis and recurrence of tumors; therefore, pinpointing mechanistic details of the pathways by which cancer cell death occurs is crucial for identifying treatment strategies and guiding clinical therapy.

Ferroptosis is defined as an iron-dependent form of nonapoptotic cell death (3), and is characterized by oxidization of polyunsaturated fatty acids (PUFAs) and accumulation of lipid peroxides. Cells undergoing ferroptosis cannot be rescued by chemical or genetic inhibitors of apoptosis or necroptosis, which indicates that ferroptosis is a distinct form of cell death (4). Ferroptosis has been investigated in many human diseases, such as neurodegenerative diseases [including Parkinson’s disease (5, 6), Alzheimer’s disease (7, 8), Friedreich’s ataxia (9), and Huntington’s disease (10)], organ injury [including hepatic damage (11, 12), brain injury (13–15), spinal cord injury (16, 17), and kidney injury (18–20)], organ fibrosis (21–23), cardiovascular diseases (24, 25), and gynecological diseases (26–28). Recently, an increasing number of studies on ferroptosis have been conducted in diverse cancers as ferroptosis is gradually being recognized as a potential form to eliminate cancer cells. In this review, we discuss the mechanism of ferroptosis and its molecular regulation in various cancers.

## Overview of Identification of Ferroptosis

In 2012, the concept of ferroptosis was first described by Scott J. Dixon (a member of the Brent R. Stockwell group) and his collaborators who described the characteristics of ferroptosis (3). Ferroptosis, a novel type of regulated cell death (RCD), is a unique form of intracellular iron-dependent peroxidation of PUFA-containing phospholipids (PLs), and is morphologically, biochemically, and genetically distinct from other forms of RCD including apoptosis, autophagy, and necroptosis. Cells undergoing ferroptosis show unique hallmarks including rupture of cellular membranes, smaller mitochondria with increased mitochondrial membrane density, reduced/vanished mitochondria cristae, rupture of outer mitochondrial membranes, and a normal nucleus (29).

### Early Studies Related to Ferroptosis

In 1955, Eagle et al. first found that human uterine carcinoma HeLa cells cultured without cystine exhibited a unique microscopic morphology that was different than that resulting from deprivation of other amino acids (30). They also found that cells cultured in cystine-free medium failed to grow but could be restored by supplementing them with glutathione (GSH) (31, 32). In 1977, Bannai et al. showed that cystine starvation of human lung fibroblasts resulted in rapid reduction of GSH and subsequent cell death; however, cell death could be rescued by the addition of the lipophilic antioxidant α-tocopherol (a component of vitamin E) (33). These results implied that cystine could sustain the intracellular level of GSH and that there might be an accumulation of reactive oxygen species (ROS) that could be prevented by lipophilic antioxidants.

In 1965, two separate research teams both identified lipid peroxidation as a prime cause of cellular damage in rat liver (34, 35). In the 1980s, lipid peroxidation was considered to be one of the main forms of oxidative damage *via* the destruction of unsaturated lipid components of cell membranes and lipoproteins in some pathologies (36, 37). Nonetheless, these discoveries were considered as mechanisms of cellular damage at that time.

### Conceptualization of Ferroptosis

Brent R. Stockwell and members tried to screen small molecules that could selectively kill cells overexpressing the oncogenic mutant *HRAS*. In 2003, they identified a novel compound that they named “erastin”, and explored the effect of erastin in engineering tumor cells. However, they found that no characteristics of apoptosis occurred, such as caspase activation, cleavage of caspase substrates, annexin V staining, and morphological changes in the nucleus (38). In 2007, they further reported that erastin induced the formation of oxidative species and subsequent death through an oxidative nonapoptotic mechanism, and that the cell death induced by erastin could be suppressed by α-tocopherol (39). In 2008, they reported yet another small compound, Ras selective lethal 3 (RSL3), which induced a similar iron-dependent non-apoptotic cell death in oncogenic RAS-harboring cancer cells, which could also be suppressed by both α-tocopherol and desferrioxamine mesylate (DFOM) (40). In 2011, the authors distinguished erastin- and RSL3-induced cell death from the mechanism of action of other cell death inducers (41). In 2012, they named this phenomenon of erastin-induced iron-dependent cell death as ferroptosis (3).

## Mechanisms of Ferroptosis

**Figure 1:**
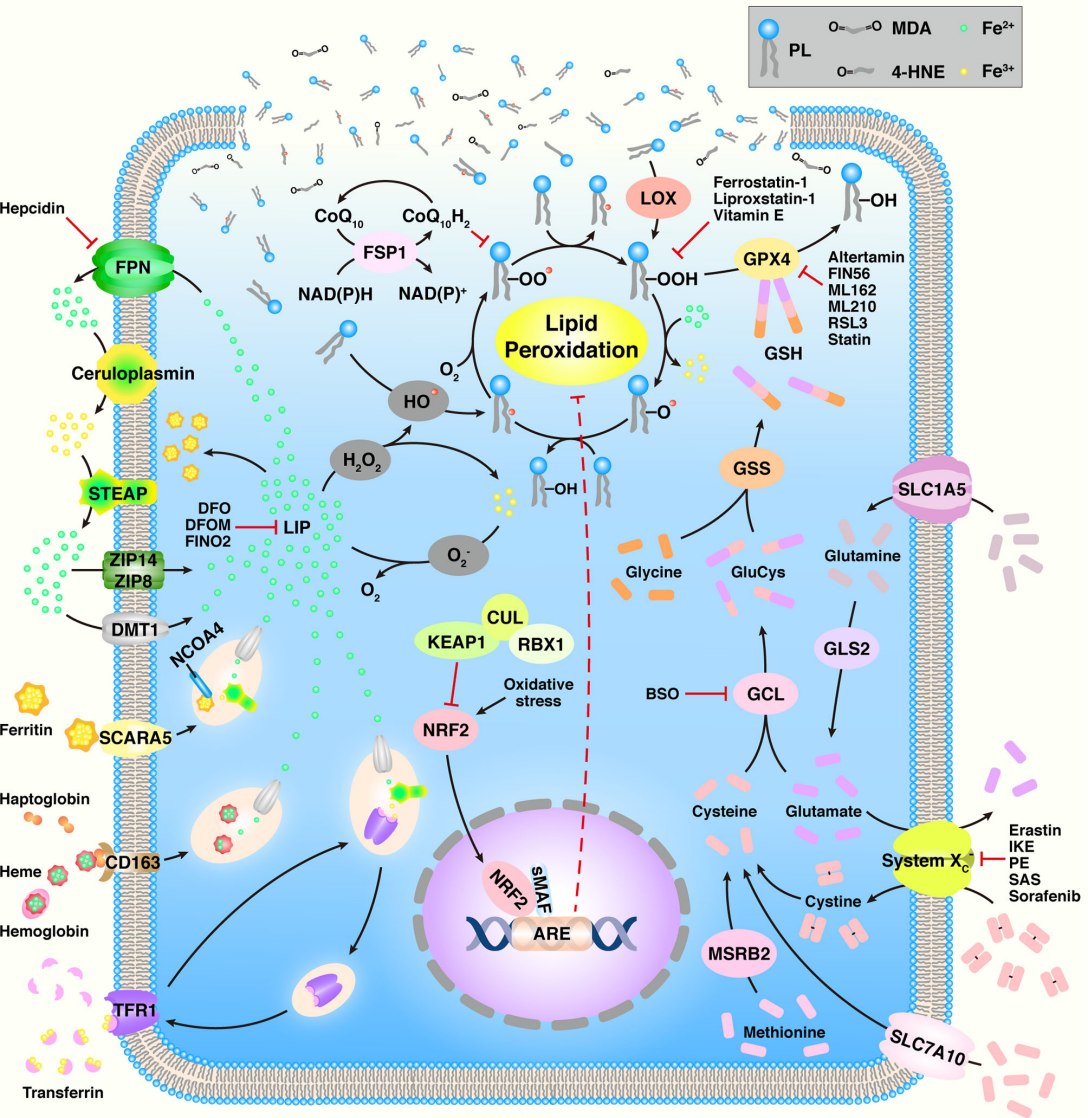
An overview of ferroptosis. ARE, antioxidant response element; BSO, buthionine sulfoximine; CoQ_10_, coenzyme Q_10_; CUL3, cullin3; DFO, deferoxamine; DFOM, desferrioxamine mesylate; DMT1, divalent metal transporter 1; FPN, ferroportin; FSP1, ferroptosis suppressor protein 1; GCL, glutamate-cysteine ligase; GLS2, glutaminase 2; GPX4, GSH peroxidase 4; GSH, glutathione; GSS, glutathione synthetase; 4-HNE, 4-hydroxynonenal; HO^•^, hydroxyl radical; IKE, imidazole ketone erastin; KEAP1, Kelch-like ECH-associated protein 1; LIP, labile iron pool; LOX, lipoxygenase; MDA, malondialdehyde; MSRB2, methionine-R-sulfide reductase B2; NCOA4, nuclear receptor coactivator 4; NRF2, nuclear factor erythroid 2-related factor 2; PE, piperazine erastin; PL, phospholipid; PL^•^, PL radical species; PLO^•^, PL alkoxyl radical; PLOH, PL alcohol; PLOO^•^, PL peroxyl radical; PLOOH, PL hydroperoxide; RBX1, ring box protein 1; RSL3, RAS synthetic lethal 3; SAS, sulfasalazine; SCARA5, scavenger receptor class A member 5; sMAF, small Maf; SLC1A5, solute carrier family member 1 member A5; STEAP, six-transmembrane epithelial antigen of prostate; TFR1, transferrin receptor 1; ZIP8, Zrt- and Irt-like protein 8.

As an increasing number of groups actively investigate ferroptosis, numerous mechanisms have been found to be involved in ferroptosis, such as amino acid metabolism, GSH metabolism, iron metabolism, and lipid metabolism (**Figure 1** and **Table 1**).

**Table 1:** Regulators of ferroptosis.

Gene	Protein	Effect	References
**Amino acid and GSH metabolism**			
***GCL***	Glutamate-cysteine ligase	Inhibition induces ferroptosis	42
***G6PD***	Glucose-6-phosphate dehydrogenase	Knockdown hinders erastin-induced ferroptosis	3
***PGD***	Phosphoglycerate dehydrogenase	Knockdown hinders erastin-induced ferroptosis	3
***SLC7A11***	Solute carrier family 7 member A11, xCT	Inhibition induces ferroptosis	3
		Knockout hinders erastin-induced ferroptosis	43
***GLS2***	Glutaminase 2	Upregulation induces p53-dependent ferroptosis	44
***GOT1***	Glutamic-oxaloacetic transaminase 1	Modulates ferroptosis in the mitochondrial tricarboxylic acid cycle	45
**Iron metabolism**			
***TFR1***	Transferrin receptor 1	Knockdown suppresses ferroptosis	46
***DMT1***	Divalent metal transporter 1	Upregulation promotes SAS-induced ferroptosis	47
***NCOA4***	Nuclear receptor coactivator 4	Knockdown suppresses ferroptosis induced by amino acid/cystine deprivation	48
***FPN***	Ferroportin	Downregulation in ferroptosis	49
**Lipid metabolism**			
***ACSL4***	Acyl-CoA synthetase long-chain family member 4	Inhibition or knockout suppresses ferroptosis	50; 51
***LPCAT3***	Lysophosphatidylcholine acyltransferase 3	Inhibition or knockout suppresses ferroptosis	50; 51
***LOX***	Lipoxygenase	Promotes ferroptosis	4
**Key regulators**			
***GPX4***	GSH peroxidase 4	Inhibition induces ferroptosis	52
***FSP1***	Ferroptosis suppressor protein 1	Induces ferroptosis resistance	53; 54
***NFE2L2***	Nuclear factor erythroid 2-related factor 2	Inhibition could reverse the resistance of cisplatin-resistant HNC cells to artesunate-induced ferroptosis	55
***TP53***	p53	Promotes ferroptosis	56; 57
		Suppresses ferroptosis	58

### Amino Acid and GSH Metabolism

GSH functions as a cofactor of GSH peroxidase 4 (GPX4, discussed in detail below) in lipid metabolism; thus, the synthesis of GSH regulates ferroptosis. GSH is synthesized with three substrates, namely glutamate, cysteine, and glycine, in two steps. The initial and limiting step is the combination of glutamate and cysteine to form GluCys in the presence of glutamate-cysteine ligase (GCL, previously known as γ-glutamyl-cysteine synthetase) utilizing ATP. Then, GluCys combines with glycine to yield GSH, which is catalyzed by GSH synthetase (GSS) in the presence of ATP. Buthionine sulfoximine (BSO) is a small molecular inhibitor of GCL known to indirectly inhibit the enzymatic activity of GPX4 (42). The pentose phosphate pathway (PPP) generates NADPH, which is essential for preserving the cellular levels of GSH. Inhibition of the PPP and silencing of two PPP enzymes, glucose-6-phosphate dehydrogenase (G6PD) and phosphoglycerate dehydrogenase (PGD), hinders erastin-induced ferroptosis in human lung cancer cells (3).

The heterodimeric amino acid antiporter system $X_{c}^{-}$ located on the cell surface consists of a twelve-pass transmembrane transporter protein solute carrier family 7 member A11 (SLC7A11, also known as xCT) and a single-pass transmembrane regulator protein SLC3A2 (4F2hc), both of which are linked by a disulfide bridge (59). It is a Na^+^-independent cystine/glutamate antiporter that imports one molecule of extracellular cystine by exporting the equivalent molecule of intracellular glutamate, followed by the conversion of the cystine to cysteine by disulfide bond breakage. Therefore, system $X_{c}^{-}$ nhibition will result in cysteine depletion in cells. In this context, several suitable compounds and their derivatives have been reported by researchers. Erastin, an oncogenic RAS-selective lethal small molecule, is the first compound reported to trigger ferroptosis by suppressing system $X_{c}^{-}$ (3). Although the precise mechanism underlying erastin-mediated inhibition of SLC7A11-mediated cysteine import remains unclear, it is agreed that erastin most likely inhibits SLC7A11 directly (60). The erastin derivatives imidazole ketone erastin (IKE) and piperazine erastin (PE) have been shown to induce ferroptosis (42, 61). Sulfasalazine (SAS), a medicine for arthritis, shows low potential inhibition of system $X_{c}^{-}$ at high concentrations (62). Sorafenib, an inhibitor of oncogenic kinases and approved by the Food and Drug Administration (FDA) as an anticancer drug, induces ferroptosis in different cancer cell lines (63–65). A high extracellular concentration of glutamate could initiate ferroptosis by preventing cystine import (3). Cysteine could also be imported into cells through the SLC7A10 transporter (66). Some cells utilize the trans-sulphuration pathway to biosynthesize cysteine from methionine through methionine-R-sulfide reductase B2 (MSRB2), that bypasses the requirement for cystine import *via* system $X_{c}^{-}$ (66, 67). Pharmacological and genetic inhibition of SLC7A11 induces ferroptosis and significantly sensitizes cisplatin-resistant head and neck cancer (HNC) cells to chemotherapy *in vitro* and *in vivo* (68). Deletion of SLC7A11 induces tumor-selective ferroptosis and inhibits pancreatic ductal adenocarcinoma (PDAC) growth (43).

Glutamine, transported into cells through SLC1A5, is also essential for the induction of ferroptotic cell death. Although glutaminase 1 (GLS1) and GLS2 are structurally and enzymatically similar and can catalyze the conversion of glutamine into glutamate, only GLS2 is required for ferroptosis (46). Upregulation of GLS2, a transcriptional target of the tumor suppressor p53, could induce p53-dependent ferroptosis (44). The glutamic-oxaloacetic transaminase 1 (GOT1) synthesizes α-ketoglutarate from glutamate, and glutaminolysis is supposed to modulate ferroptosis in the mitochondrial tricarboxylic acid (TCA) cycle (45).

### Iron Metabolism

The first observation that iron participates in the process of ferroptosis originated from reports that iron chelators could prevent cell death induced by cystine deprivation (considered as ferroptosis now) in 1996 (69). The biological function of iron is mainly dependent on its ability to accept and donate electrons while switching among the ferrous bivalent (Fe^2+^), ferric trivalent (Fe^3+^), and ferryl tetravalent (Fe^4+^) states by catalyzing various biochemical reactions (70). At the cellular level, three forms of ROS, including superoxide $\left(O_{2}^{-}\right)$, hydrogen peroxide (H_2_O_2_), and hydroxyl radical (HO^•^), are related to iron in redox reactions. $O_{2}^{-}$ and H_2_O_2_ are respectively known as single and double univalent reductions of molecular oxygen (O_2_) respectively, while the most chemically active ROS is HO^•^, which can cause nonspecific oxidation that destroys biological molecules such as lipids. In the Fenton reaction, Fe^2+^ is oxidized to Fe^3+^ by a reaction with H_2_O_2_, while the electron transfers to H_2_O_2_ to form HO^•^ (71). Conversely, Fe^3+^ could be reduced back to Fe^2+^ through reaction with $O_{2}^{-}$ while $O_{2}^{-}$ loses an electron to form O_2_, which is known as the Haber-Weiss reaction (72).

Sufficient levels of free intracellular iron is a necessity for triggering ferroptosis. There are four following routes for cells to import iron (73). The most crucial route is mediated by transferrin and its partner, the transferrin receptor 1 (TFR1). Transferrin, a type of globulin mainly synthesized by the liver and released into the serum, has an excellent ability to chelate two Fe^3+^ ions. When the transferrin reaches the cell membrane, it identifies and binds to TFR1 and the transferrin-TFR1 complex is internalized *via* clathrin-mediated endocytosis, whereas apo-transferrin (iron-free transferrin) is not identified by TFR1 and internalized. Fe^3+^ is liberated from the transferrin-TFR1 complex at the low pH conditions of endocytic vesicles and reduced to Fe^2+^ by the six-transmembrane epithelial antigen of prostate (STEAP) family (73, 74). Fe^2+^ is then released into the cytoplasm *via* the divalent metal transporter 1 (DMT1) and participates in establishing the intracellular labile iron pool (LIP) (75). Meanwhile, apo-transferrin and TFR1 remain bound until the complex is recycled back to the plasma membrane, followed by apo-transferrin releases (76). The second route is directly assimilating the free iron unbound by transferrin; however, each iron transporter only transports Fe^2+^. Therefore, Fe^3+^ is first reduced to Fe^2+^ by several types of ferrireductases (*e.g.*, STEAP, cytochrome B reductase 1, and ferric chelate reductase 1), and the Fe^2+^ is directly transported into cells by cellular membrane transporters including DMT1, Zrt- and Irt-like protein 8 (ZIP8), and ZIP14 (73). The third pathway involves the uptake of hemoglobin-containing porphyrin-bound Fe^2+^, especially in macrophages (77). The fourth mechanism involves the assimilation of the iron-storage protein ferritin by its cellular membrane receptors such as the scavenger receptor class A member 5 (SCARA5) found in embryos and in the kidney (78).

After iron uptake, the intracellular iron can be utilized, stored, and exported. In terms of utilization, mitoferrin 1 and 2 specially transport iron into the mitochondria to assist with cellular respiration and synthesis of Fe-S clusters and heme (79). There are two forms of intracellular storage, LIP and ferritin. LIP causes active oxidative stress-related toxicity and is responsible for regulating cellular iron homeostasis through the iron regulatory protein-iron responsive element system. Ferritin is an iron-sequestering protein that contains up to 4500 iron atoms and possesses multiple functions in iron delivery, cell proliferation, angiogenesis, and immunosuppression (80). Ferritin can also be degraded to release free iron *via* the nuclear receptor coactivator 4 (NCOA4), a process termed as “ferritinophagy” (81). In terms of iron export, ferroportin (FPN, *i.e.*, SLC40A1), once known as the only iron efflux pump that cooperates with ceruloplasmin or hephaestin, is mainly responsible for transporting Fe^2+^ out of cells (82). Ceruloplasmin suppresses ferroptosis by regulating iron homeostasis in HepG2 and Hep3B cells, and depletion of ceruloplasmin results in the accumulation of intracellular Fe^2+^ and lipid ROS and promotes erastin- and RSL3-induced ferroptotic cell death (83). In addition, prominin 2 promotes the formation of ferritin-containing multivesicular bodies and exosomes that transport iron out of the cell to facilitate ferroptosis resistance in breast cancer (84).

Treating cells without transferrin or with TFR RNAi could not lead to significant ferroptotic cell death, indicating that an external iron source is required for ferroptosis (46). STEAP1 and STEAP2 are highly expressed in various human cancer types, such as the colon, breast, cervix, prostate, pancreas, bladder, ovary, testis, and Ewing sarcoma (85–87). STEAP3 is overexpressed in malignant gliomas and induces cancer epithelial–mesenchymal transition (EMT) (88). STEAP4 is activated under hypoxic conditions, leading to mitochondrial iron imbalance and enhanced ROS production (89). FPN is dramatically suppressed in many cancer types (90), indicating that there might be abundant iron in cancer cells. Reduced FPN levels in triple-negative breast cancer (TNBC) cells stimulate proliferation and EMT (91). Hepcidin, synthesized by tumors or the liver, facilitates FPN degradation and contributes to cancer proliferation and progression (92). Proteins involved in raising the levels of intracellular iron (TFR1, DMT1, and hepcidin) are extensively upregulated in tumor cells, whereas proteins suppressing intracellular iron accumulation (FPN and hephaestin) are downregulated (93).

### Lipid Metabolism

The fact that ferroptosis is driven by peroxidation of PUFAs, and not monounsaturated fatty acids (MUFAs) or deuterated PUFAs, was confirmed by Brent R. Stockwell and coworkers in 2016 (4). As is known to all, PUFAs, as well as essential fatty acids, could not be physiologically synthesized and must be obtained from food. PUFA is defined as a fatty acid containing at least two -CH=CH- groups, such as linoleic acid, linolenic acid, and arachidonic acid (AA). PLs, the basic components of the cellular membrane, are classified as glycerophospholipids and sphingomyelin. The predominant fatty acid moiety in sphingomyelin is saturated fatty acids or MUFAs, whereas glycerophospholipids contain an esterified C_16_ or C_18_ saturated fatty acid at C-1, an esterified C_18_ to C_20_ unsaturated fatty acid (*e.g.*, AA) at C-2, and a phosphatidic ester group at C-3 of the glycerol backbone. The incorporation of *de novo* synthesized fatty acids into PLs requires acyl-CoA synthetases (ACSs) that convert long-chain fatty acids to acyl-CoA and lysophospholipid acyltransferases (LPLATs) that, in turn, catalyze the subsequent reacylation step to form PLs (94). For instance, lipidomic studies suggest that phosphatidylethanolamines containing AA or adrenic acid (AdA), are key PLs that undergo oxidation and drive the occurrence of ferroptosis (50, 51). ACS long-chain family member 4 (ACSL4) preferentially converts AA to acylated AA, and lysophosphatidylcholine acyltransferase 3 (LPCAT3) subsequently catalyzes the incorporation of acylated AA into the PL (51, 95).

There are two mechanisms of lipid peroxidation, the non-enzymatic free radical chain reaction and the enzymatic process. Compared with the tightly controlled enzymatic lipid peroxidation, the free radical chain reaction involving Fenton chemistry is poorly controlled. There are three types of lipid oxidation enzymes, namely cyclooxygenase (COX), cytochrome P450 (CYP), and lipoxygenase (LOX). COXs synthesize lipid endoperoxides and are partially responsible for the peroxidation of linoleic acid, while CYPs synthesize epoxyeicosatrienoic acids and LOXs contribute predominantly to the synthesis of lipid hydroperoxides.

Nonenzymatic lipid peroxidation involves the oxidation of PUFAs, initiated by ROS. In unsaturated acyl chains, the allylic hydrogen atoms, *i.e.*, the hydrogen atoms on methylene groups adjacent to double bonds, exhibit low carbon-hydrogen (C-H) bond energies, while the hydrogen atoms located on methylene between two double bonds (bis-allylic hydrogen atoms) have even lower C-H bond energies, thus bis-allylic hydrogen atoms can be abstracted by ROS, forming PL radical species (PL^•^) with the radical centered on the allylic carbon atom (96). Therefore, PUFAs are highly susceptible to oxidative damage due to the existence of bis-allylic hydrogen atoms that saturated fatty acids and MUFAs do not possess. According to previous reports (96, 97), in the presence of HO^•^ derived from the Fenton reaction, the PUFA of PL donates a hydrogen atom to HO^•^ and becomes a carbon-centered PL^•^ that further reacts with intracellular molecular O_2_ to form a PL peroxyl radical (PLOO^•^). Next, with the participation of PUFA moiety from another PL, PLOO^•^ abstracts a hydrogen atom and subsequently converts to PL hydroperoxide (PLOOH) accompanied with a new PL^•^. PLOOH is cleaved in the presence of Fe^2+^ to form the PL alkoxyl radical (PLO^•^) that reacts with PUFA of another PL to form PL alcohol (PLOH) and a new PL^•^, followed by another lipid radical chain reaction.

The enzymatic pathway mainly involves iron-containing LOXs, which can catalyze the site-specific oxidation of PUFAs in a controlled manner. LOXs are classified based on their regioselectivity (*e.g.*, the number of carbon atoms subjected to deoxygenation) and stereoselectivity (“S” or “R”) (98). There are six LOX isoforms discovered in humans, namely 5-LOX, 12-LOX, 12R-LOX, 15-LOX-1, 15-LOX-2, and e-LOX3, ranging from 662 to 711 amino acids and sharing 44% of their sequence identity (99). Encoded by the *ALOX5* gene, 5-LOX could oxide AA at C-5 to form 5-hydroperoxyeicosatetraenoic acid (5-HpETE), which is subsequently converted to leukotriene A_4_ (LTA_4_) by 5-LOX (100, 101). Once 5-LOX is activated, it migrates to the nuclear membrane where it associates with two additional proteins: the 5-LOX activating protein (FLAP) and cytosolic phospholipase A2 (cPLA2) (102). cPLA2 is responsible for cleaving AA from PLs to increase substrate availability for 5-LOX. Although the exact function of FLAP is still unclear, pharmacological inhibition of FLAP function prevents oxidation of endogenous AA by 5-LOX, which demonstrates the necessary role of FLAP in lipid peroxidation (103). Compared with 5-LOX, which oxidizes hydrolyzed arachidonoyl groups of PLs by cPLA2, 12-LOXs and 15-LOXs, encoded by *ALOX12/ALOX12B* and *ALOX15/ALOX15B*, respectively, directly oxidize AA to synthesize 12-HpETE and 15-HpETE, which are rapidly reduced to their corresponding hydroxides and hepoxilins (104). Meanwhile, 12-LOXs and 15-LOXs can also oxidize linoleic acid to generate 13-hydroperoxyoctadecadienoic acid (13-HpODE), which is subsequently reduced to 13-HODE (101). Docosahexanoic acid (DHA) is also a substrate for 15-LOX-1, which metabolizes the conversion of ω-3 fatty acid to 17-hydroperoxydocosahexaenoic acid (17-HpDHA), which is rapidly transformed into resolvins and protectins (105).

Lipid peroxidation of membranes substantially alters the physical properties of lipid bilayers in terms of disrupted ion gradients, decreased membrane fluidity, slower lateral diffusion, and increased membrane permeability (106). There exist two mechanisms for eliminating lipid peroxides from cells without the generation of new radicals. GPXs, especially GPX4, are crucial regulators for reducing lipid peroxides to corresponding alcohols by utilizing GSH as a cofactor (107), thereby limiting the transition metal-dependent formation of toxic radicals (*e.g.*, PLO^•^). Another mode of degradation involves the conversion of lipid peroxides to aldehydes such as malondialdehyde (MDA) and 4-hydroxynonenal (4-HNE), which should undergo multiple oxidization steps. However, there is still no definitive consensus about the metabolic route that predominantly contributes to the degradation of lipid peroxides among multiple biosynthesis routes (101). MDA could react with primary amines on proteins or DNA to form crosslinks. Moreover, excessive MDA generation within cells is associated with major human diseases including cancer (108). Decomposition of AAs and longer PUFAs generates 4-HNE, which contains three functional groups: (i) an electrophilic C=C double bond that is a Michael acceptor and that forms covalent adducts with nucleophilic amino acids; (ii) an aldehyde that can form Schiff base adducts with primary amines; and (iii) a hydroxyl group that can be oxidized to an electrophilic ketone (109). The electrophile 4-HNE has been widely studied as a signaling molecule that stimulates the cell cycle and cellular proliferation, along with investigation into its cytotoxic nature known to inhibit gene expression (110).

### Key Regulators of Ferroptosis

#### GPX4

According to phylogeny, the GPX family is divided into three groups: GPX1 and GPX2; GPX3, GPX5, and GPX6; GPX4, GPX7, and GPX8, and could also be classified as selenocysteine-containing GPXs and cysteine-containing GPXs depending on whether the active center contains Sec or Cys (111). GPX4, *i.e.*, PL hydroperoxide GPX, is the second selenoperoxidase to be isolated and is regarded as a peroxidation-inhibiting protein after the identification of GPX1 (112). GPX4 is the only GPX for which PL hydroperoxides in membranes and protein-thiol groups can act as oxidizing and reducing substrates, respectively, under conditions of GSH deprivation (113). It was first isolated and purified from pig liver in 1982, and was identified to confer protection to liposomes and biomembranes from iron-catalyzed lipid peroxidation (114). The human GPX4 was isolated from human liver in 1994 (115). GPX4, containing 170 amino acids, has a typical thioredoxin motif consisting of four α-helices that are localized at the protein surface and seven β-strands, five of which form a central β-sheet (116). The catalytic triad, consisting of selenocysteine (U46), glutamine (Q81), and tryptophan (W136) residues, is localized at the protein surface, and mutations in any of these residues could lead to the inactivation of GPX4 (117). For instance, replacement of selenocysteine with cysteine could diminish the activity of GPX4 by 90% and strengthen its sensitivity to redox stress (118). GPX4 reduces lipid peroxides to the corresponding alcohols *via* oxidation of its active site selenol (Se-H) to selenenic acid (Se-OH), which is then reduced by two equivalents of GSH to the active Se-H (101).

RSL3 has four diastereomers, but only 1S,3R-RSL3 can induce ferroptosis by suppressing the enzymatic activity of GPX4 *via* covalent modifications without affecting intracellular GSH levels (42, 119). Conversely, overexpression of GPX4 could prevent RSL3-induced ferroptotic cell death (42). RSL3 induces ferroptosis in colorectal cancer cells due to decreased expression of GPX4 and both increased levels of ROS and cellular LIP (52). In addition to RSL3, ML162, ML210, and altretamine (an FDA-approved anticancer agent) can also induce ferroptosis by suppressing GPX4 (120). Inducible GPX4 inactivation could also lead to 12/15-LOX-derived lipid peroxidation as a specific downstream event, which indicates that GPX4 could prevent cell death by inhibiting 12/15-LOX (113). FIN56, derived from CIL56, exhibits greater potency and better oncogenic RAS selectivity than CIL56 (121). FIN56 induces ferroptosis by degrading GPX4 protein, instead of downregulating GPX4 mRNA transcription and subsequent synthesis of GPX4 protein, and simultaneously causes depletion of mevalonate-derived coenzyme Q_10_ (CoQ_10_), which is an electron carrier in the mitochondrial respiratory chain and an endogenous antioxidant. FINO2, an endoperoxide-containing 1,2-dioxolane, can initiate ferroptosis selectively in engineered cancer cells, such as renal cancer cell Caki-1 and fibrosarcoma cell HT-1080 (122). FINO2 does not inhibit the antiporter system $X_{c}^{-}$; instead, it directly represses the enzymatic activity of GPX4, or depletes GPX4 and CoQ_10_. However, it can directly oxidize iron and indirectly suppresses the enzymatic function of GPX4, ultimately causing widespread lipid peroxidation. The side effects of statin treatment could be attributed to reduced levels of GPX4 in some tissues (123), while the decreased incidence of several cancers on statin consumption (124) and the loss of GPX4 detected in some cancer cells with statin treatment could be ascribed to repression of GPX4 synthesis (125). Further, statin treatment has also been shown to deplete CoQ_10_ (126).

#### FSP1

Ferroptosis suppressor protein 1 (FSP1), previously known as apoptosis-inducing factor mitochondrial-associated 2 (AIFM2), was recently identified as a ferroptosis resistance factor (54). Although GPX4 is essential for cancer cells to escape from ferroptosis, inhibition of GPX4 fails to trigger ferroptosis in some cancer cells regardless of ACSL4 expression, suggesting the existence of additional mechanisms of ferroptosis resistance. The anti-ferroptotic function of FSP1 is independent of cellular GSH level, GPX4 activity, ACSL4 expression, and oxidizable fatty acid content, showing that FSP1 does not interfere with canonical ferroptosis mechanisms. Moreover, p53 status does not affect FSP1 expression. FSP1 expression is positively correlated with ferroptosis resistance in many cancer cell lines (127). FSP1 can prevent PL peroxidation and its suppression has been shown to be enhanced in the presence of both CoQ_10_ and α-tocopherol (54). In the presence of FSP1, CoQ_10_ was reduced to CoQ_10_-H_2_ using NADPH to inhibit the propagation of PL peroxidation. Inhibition of FSP1 can robustly sensitize cells to RSL3-induced ferroptosis (53, 54).

#### NRF2

Nuclear factor erythroid 2-related factor 2 (NRF2) encoded by *NFE2L2* is a transcription factor that consists of 605 amino acids and contains seven conserved homology domains termed as Neh1-Neh7 (128, 129). Neh2 is the major regulatory domain that has two binding motifs known as DLG and ETGE, both of which help to regulate NRF2 stability by interacting with the Kelch domains of the E3 ubiquitin ligase Kelch-like ECH-associated protein 1 (KEAP1), which is the most notable negative regulator of NRF2 and contains a number of cysteine residues (including C151, C273, and C288) that act as electrophilic attack centers (130). Under physiological conditions, NRF2 is maintained at low levels in normal tissues because of KEAP1-dependent ubiquitination (130). KEAP1 acts as an adaptor for NRF2 binding to the KEAP1-cullin 3-ring box protein 1 (KEAP1-CUL3-RBX1) E3 ubiquitin ligase complex, which targets NRF2 for rapid proteasomal degradation (129). During increased oxidative stress, or if KEAP1, CUL3, or NRF2 are mutated, NRF2 can no longer be ubiquitylated and degraded, and the newly translated NRF2 transfers to the nucleus and activates transcription of anti-oxidant response element (ARE)-containing genes, many of which play a key role in preventing the initiation of ferroptosis (131). RSL3 or ML-162 treatment increases the expression of p62 and NRF2 in chemo-resistant HN3R and HN3-rslR cells, inactivates KEAP1, and increases the expression of the phospho-protein kinase R-like endoplasmic reticulum kinase (p-PERK)-activating transcription factor 4 (ATF4)-sertrin 2 (SESN2) (132). NRF2 inhibition could reverse the resistance of cisplatin-resistant HNC cells to artesunate-induced ferroptosis (55). With regard to other Neh domains, Neh1 known as a DNA binding domain enhances NRF2 transcriptional activation (133). The Neh3, Neh4, and Neh5 domains are known as trans-activation domains of NRF2 (134–136). Neh6, a serine-rich domain, negatively modulates NRF2 stability (137), and Neh7 has been shown to interact with the nuclear receptor retinoid X receptor α (RXRα), which inhibits the transcription of NRF2 target genes (138).

The genetic products of NRF2 activation can be categorized functionally with respect to their involvement in three cellular processes consisting of iron/metal metabolism, intermediate metabolism, and GSH synthesis/metabolism. Resistant melanoma cells can efficiently activate NRF2 (which upregulates the early ferroptotic marker GSH-specific γ-glutamylcyclotransferase 1 (CHAC1) in an endoplasmic reticulum (ER) stress-independent manner) and aldo-keto reductase AKR1C1-3 (which degrades lipid peroxides generated by 12/15-LOX), which could cumulatively result in resistance to ferroptotic cell death (139). NRF2 targets many genes involved in iron/metal metabolism, such as the light chain and heavy chain of ferritin (*FTL/FTH1*), *FPN*, heme oxygenase-1 (*HO-1*), biliverdin reductase A and B (*BLVRA/B*), ferrochelatase (*FECH*), ATP-binding cassette sub-family B member 6 (*ABCB6*), and *SLC48A1*, which are all regulated by NRF2 (140–145). In terms of the relationship between NRF2 and intermediate metabolism, NRF2 targets are involved in lipid metabolism (*i.e.*, peroxisome proliferator-activated receptor γ, *PPARG*), the reduction of aldehydes and ketones to their alcohol forms (*i.e.*, aldo-keto reductases, *AKR1C1–3*, *AKR1B1*, and *AKR1B10*), and glucose metabolism/NAPDH regeneration (*i.e.*, *G6PD*) (146–149). Considering GSH synthesis/metabolism, numerous processes are under the control of NRF2, such as GSH metabolism (*i.e.*, *GCL*, *GSS*, and *SLC7A11*), redox (*i.e.*, GSH-S-transferases pi 1 and α 1, *GSTP1* and *GSTA1*; peroxiredoxin 1 and 6, *PRDX1* and *PRDX6*; and thioredoxin reductase, *TXNRD*), and the reduction of lipid peroxides (*i.e.*, *GPX4*) (150–152). As mentioned above, some of these progresses have been confirmed to be involved in ferroptosis.

#### p53

The tumor suppressor gene *TP53*, regarded as the “guardian of the genome”, was first discovered in 1979 in a complex with the simian virus 40 large T antigen and has been widely studied in cancers (153). Many studies have suggested that p53 could act synergistically with established oncogenes to promote the conversion of normal tissues to tumors and lead to accelerated metastasis. The first evidence that p53 might act as a tumor suppressor was reported in 1984 (154). Subsequently, it was concluded that these previous experiments were performed not with wild-type p53, but with its mutated version (155). The mutant p53 is highly abundant in cancers, and it promotes tumorigenesis by disabling the function of the wild-type p53, as well as by “gain-of-function” processes such as the accumulation of p53 mutations, that augments the oncogenic capacity of the mutated p53 and delivers a stronger tumoral resistance against anti-cancer treatments (156, 157). Under normal conditions, the levels of p53 are low because of the E3 ubiquitin-protein ligase MDM2, which can target and degrade p53, while oncogene activation could prevent MDM2 binding to p53 and stimulate p53 acetylation (158). Although 42% of cases across 12 tumor types occur due to *TP53* mutation, the rate varies widely across diverse types of cancers (159).

Apart from the effects of p53 on apoptosis, autophagy, and the cell cycle, it also regulates ferroptosis *via* transcriptional or post-transcriptional mechanisms. Intriguingly, p53 can enhance or inhibit ferroptosis *via* different pathways. As to pro-ferroptosis, p53 promotes ferroptosis of MCF7 and U2OS cells due to SLC7A11 repression without affecting the expression of other p53 target genes involved in the cell cycle and apoptosis (56). p53 targets spermidine/spermine N1-acetyltransferase 1 (SAT1), which correlates with the expression of 15-LOX, and promotes the expression of SAT1 to induce ferroptosis (57). Depletion of p53 prevents nuclear accumulation of dipeptidyl peptidase-4 (DPP4) and triggers membrane-associated DPP4-mediated lipid peroxidation by binding to NADPH oxidase 1 (NOX1), which culminates in the ferroptosis of colorectal cancer cells. On the other hand, p53 mediates expression of the tumor suppressor cyclin-dependent kinase inhibitor 1A (CDKN1A/p21), which is a key mediator of p53-dependent cell cycle arrest after DNA damage, delays the onset of ferroptosis in response to cystine deprivation in cancer cells, and increases p53 expression by using the MDM2 inhibitor nutlin-3 that blocks erastin-induced ferroptosis in HT-1080 cells (58).

## Ferroptosis and Cancer

Here, different cancer groups are discussed in the context of ferroptosis, and the numerous inducers and inhibitors of ferroptosis, including drugs, genes, and RNA are listed in **Table 2**.

**Table 2:** Inducers and inhibitors of ferroptosis in different cancers.

Cancer	Inducer	Reference	Inhibitor	Reference
**Hepatocellular carcinoma**	Auranofin BSO Haloperidol Rb miRNA-214-3p	160; 161; 162; 163	Ceruloplasmin S1R CISD1 CBS O-GlcNAcylated c-Jun lncRNA GABPB1-AS1	164; 165; 166; 83; 167; 168
**Pancreatic cancer**	Artesunate Ruscogenin ARF6 Piperlongumine STAT3	169; 170; 49; 171; 172	LONP1 KRAS^G12D^ GPR78	173; 174; 175
**Glioma**	Dihydroartemisinin Amentoflavone Pseudolaric acid B	176; 177; 178	HSPA5 GDF15 circRNA TTBK2	176; 179; 180
**Lung cancer**	Acetaminophen Erianin	181; 145	NFS1 STYK1 EGLN1/c-Myc lncRNA LINC00336	182; 183; 184; 185
**Breast cancer**	SAS Siramesine Lapatinib Dihydroisotanshinone I Ferroptocide	186; 187; 188; 47	–	–
**Head and neck cancer**	RSL3 ML-162 Dihydroartemisinin	189; 132	SLC7A11 CISD2	190; 68
**Colorectal cancer**	RSL3 Bromelain Birch Etna Vitamin C	191; 192; 52	–	–
**Gastric cancer**	ACP PG	193; 194	miRNA-522 miRNA-103a-3p circRNA 0008035	195; 196; 194
**Melanoma**	miRNA-9	197	AKR1C1-3 Nedd4 miRNA-137,	139; 198; 199
**Leukemia**	Dihydroartemisinin Typhaneoside HMGB1	200; 201; 202	–	–
**Ovarian cancer**	TAZ	203	SCD1 CBS	204; 205
**Renal cell carcinoma**	HIF-2α TAZ MAPK	206; 207; 208	*VHL*	209
**Lymphoma**	Artesunate	210	–	–
**Osteosarcoma**	Phenethyl isothiocyanate	211	–	–
**Esophageal cancer**	DNAJB6	212	–	–

ACP, Actinidia chinensis Planch; AKR1-3, aldo-keto reductase 1-3; ARF6, ADP-ribosylation factor 6; BSO, buthionine sulfoximine; CBS, cystathionine β-synthase; CDO1, cysteine dioxygenase 1; CISD1, CDGSH iron sulfur domain 1; DNAJB6, DNAJ/Hsp40 homolog subfamily B member 6; GDF15, growth/differentiation factor 15; GPR78, glucose-regulated protein 78; HIF-2α, hypoxia inducible factor 2α; HMGB1, high mobility group box 1; HSPA5, heat shock protein family A member 5; IKE, imidazole ketone erastin; LONP1, lon peptidase 1; MAPK, mitogen-activated protein kinase; NFS1, human mitochondrial cysteine desulfurase; PG, physcion 8-O-β-glucopyranoside; Rb, retinoblastoma; S1R, Sigma-1 receptor; SAS, sulfasalazine; SCD1, steroyl-CoA desaturase 1; SLC7A11, solute carrier family 7 member A11; STAT3, signal transducer and activator of transcription 3; STYK1, serine threonine tyrosine kinase 1; TAZ, transcription regulator 1; VHL, vov Hippel-Lindau.

### Pharmaceuticals-Induced Ferroptosis

Many clinical drugs have the ability to induce ferroptosis in cancer cells, which means that researchers need to comprehensively explore the potential mechanisms of these drugs. Artesunate, an anti-malarial drug, can specifically induce ferroptosis in pancreatic cancer cells without affecting pancreatic ductal epithelial cells (169). Artesunate also activates the *ATF4-CHOP-CHAC1* cascade in DAUDI and CA-46 cells, and CHAC1 enhances artesunate-induced ferroptosis in Burkitt’s lymphoma cells (210). Dihydroartemisinin, a semisynthetic derivative of artemisinin, increases ROS levels in a dose-dependent manner and decreases the levels of both GPX4 and RAS in HEP-2 and CNE-1 cells (189). Dihydroartemisinin can also regulate the activity of the AMP-activated protein kinase (AMPK)/mTOR/p70S6k signaling pathway, thereby accelerating ferritin degradation, increasing LIP, promoting cellular ROS accumulation, and eventually triggering ferroptosis in acute myeloid leukemia cells (200). Several anti-inflammatory drugs have shown the potential to induce ferroptosis. Combinatorial treatment of auranofin and BSO can downregulate GPX4 and accumulate NRF2 and HO-1 (162). Ibuprofen induces ferroptosis in glioblastoma cells by downregulating the NRF2 signaling pathway (213). Acetaminophen can strengthen the sensitivity of erastin-induced ferroptosis by modulating the NRF2/HO-1 signaling pathway in non-small-cell lung cancer (NSCLC) (145). SAS can induce ferroptosis in breast cancer cells by upregulating TFR1 and DMT1, especially in cells with low expression of estrogen receptors (47). The tranquilizer haloperidol promotes ferroptosis by accelerating iron accumulation, lipid peroxidation, and GSH depletion under erastin or sorafenib treatment in HepG2 and Huh-7 cells (160). The lysosome disruptor siramesine and the tyrosine kinase inhibitor lapatinib synergistically induce ferroptosis by increasing the expression of transferrin and decreasing the expression of FPN (188).

Many extracts from plants and herbs also exhibit anti-tumor effects by inducing ferroptosis. Ruscogenin can induce ferroptosis by improving ROS generation and trigger intracellular iron accumulation by upregulating transferrin and downregulating FPN (49). Piperlongumine can also induce ferroptosis in pancreatic cancer cells (171). Amentoflavone represses the expression of FTH by autophagy *via* activation of the AMPK/mTOR/p70S6K signaling pathway to trigger *in vitro* and *in vivo* ferroptosis in an autophagy-dependent manner (177). Typhaneoside, a major flavonoid found in the extract of *Pollen Typhae*, promotes the activation of the AMPK signaling pathway to contribute to ferritin degradation, ROS accumulation, and ferroptosis in Kas-1, HL-60, and NB4 cells (202). Pseudolaric acid B isolated from cortex pseudolaricis triggers ferroptosis *in vivo* and *in vitro* by upregulating TFR1, activating NOX4, and inhibiting SLC7A11 (178). Actinidia chinensis Planch (ACP) has been shown to increase the accumulation of ROS by inhibiting GPX4 and SLC7A11 to induce ferroptosis in HGC-27 cells (193). Dihydroisotanshinone I, a pure compound present in danshen, can induce ferroptosis by downregulating the expression of GPX4 both *in vitro* and *in vivo* (186). The extract of *Betula etnensis* Raf. (Birch Etna) promotes an oxidative cellular microenvironment resulting in HO-1-mediated ferroptosis of CaCo-2 cells (191). Erianin, a natural product isolated from *Dendrobium chrysotoxum Lindl*, induces ferroptotic cell death in lung cancer cells *via* Ca^2+^/calmodulin signaling (181).

Some new compounds are still being found by researchers. The novel compound ferroptocide, identified as an inhibitor of thioredoxin, could rapidly and robustly induce ferroptotic cell death and positively modulate the immune system in a breast cancer model (187). Phenethyl isothiocyanate, present in cruciferous vegetables, induces ferroptosis, autophagy, and apoptosis in K7M2 cells by activating the ROS-related mitogen-activated protein kinase (MAPK) signaling pathway (211).

### Gene and Protein-Regulated Ferroptosis

#### Positive Regulators

Overexpression of some genes and proteins can promote ferroptosis in cancer cells. The level of DNAJ/Hsp40 homolog subfamily B member 6 (DNAJB6) is negatively correlated with lymph node metastasis in esophageal squamous cell carcinoma (ESCC) patients, and the overexpression of its isoform DNAJB6a is accompanied by remarkable reduction in the protein levels of GPX4 and phospho-AKT (p-AKT), thus DNAJB6a plays an anti-oncogenic role in ESCC progression *via* ferroptosis (212). Signal transducer and activator of transcription-3 (STAT3) can promote ferroptosis through activation of cathepsin B-mediated lysosomal cell death in PANC-1 and CFPAC-1 cells (170).

Loss of function of genes and proteins could change the sensitivity of cancer cells to ferroptosis. The loss of function of retinoblastoma (Rb) protein is significant during liver carcinogenesis, and the Rb-negative status of hepatocellular carcinoma (HCC) cells promotes ferroptosis on sorafenib treatment (163). Knockdown of high mobility group box 1 (HMGB1) decreases erastin-induced ROS generation *via* an iron-mediated lysosomal pathway in HL-60 cells expressing NRAS^Q61L^ (201).

Some genes and proteins can affect lipid metabolism and subsequently induce ferroptosis. Bromelain induces ferroptosis by inhibiting the proliferation of KRAS mutant colorectal cancer cells *via* ACSL4 (192). ADP-ribosylation factor 6 (ARF6) can sensitize gemcitabine-resistance pancreatic cancer cells to RSL3-induced lipid peroxidation by affecting the ACSL4 protein levels (172). Hypoxia-inducible transcriptional factor 2α (HIF-2α) selectively enriches PUFAs by activating the expression of the hypoxia-inducible lipid droplet-associated (HILPDA) protein and induces a ferroptosis-susceptible cell state in 786-O cells (208).

Several cellular signaling pathways are also involved in ferroptosis. Removal of the Hippo pathway effector transcription regulator 1 (TAZ) confers ferroptosis resistance, whereas overexpression of TAZS89A sensitizes cells to ferroptosis. TAZ promotes ferroptosis by regulating epithelial membrane protein 1 (EMP1) and NOX4 in renal cell carcinoma (207). Low levels of TAZ in chemo-resistant recurrent ovarian cancer are responsible for reduced ferroptosis susceptibility (203). Blockade of MAPK signaling similarly protects cells from ferroptosis, whereas NSCLC with sustained MAPK activation is likely to respond to ferroptosis following cystine depletion (206).

#### Negative Regulators

Many genes and proteins can protect cancer cells from ferroptosis. The sigma-1 receptor (S1R) protects HCC cells against sorafenib, while knockdown of S1R could induce ferroptosis by blocking the expression of GPX4 (164). The human mitochondrial cysteine desulfurase (NFS1) is highly expressed in well-differentiated lung adenocarcinoma and protects cells from ferroptosis (182). CDGSH iron sulfur domain 1 (CISD1), an iron-containing outer mitochondrial membrane protein, negatively regulates erastin-induced ferroptosis in HepG2 and Hep3B cells by inhibition of mitochondrial lipid peroxidation in a GPX4-independent manner (168). Overexpression of the *CISD2* gene has been shown to confer resistance to HN-6 and HN-10 cells against SAS-induced ferroptosis, while silencing *CISD2* could reverse SAS-resistant cells to a ferroptotic state with increased levels of lipid ROS and mitochondrial Fe^2+^ (190).

Some genes and proteins hinder ferroptosis; however, ferroptosis can occur when these are suppressed. NRF2 can upregulate cystathionine β-synthase (CBS) to confer resistance to erastin-induced ferroptosis in ovarian cancer cells (204), however inhibition of CBS can trigger ferroptosis in HCC (167). Inhibition of Lon peptidase 1 (LONP1) could contribute to ferroptosis of PANC-1 cells by activating the NRF2/KEAP1 signaling pathway and upregulating GPX4 expression (175). Heat shock protein family A member 5 (HSPA5) can upregulate the expression and activity of GPX4 to prevent dihydroartemisinin-induced ferroptosis in glioma (176). Inhibition of glucose-regulated protein 78 (GRP78), one of the most active molecular chaperone components in the ER of cancer cells, could enhance the effect of artesunate-induced ferroptosis in *KRAS* mutant pancreatic cancer cells (174). Growth/differentiation factor 15 (GDF15) knockdown has been shown to promote erastin-induced ferroptosis in MGC803 cells by attenuating SLC7A11 expression and subsequently decreasing intracellular GSH levels (179).

Some genes and proteins can prevent ferroptosis *via* affecting lipid metabolism. EGLN1/c-Myc directly activates the expression of lymphoid-specific helicase (LSH) by inhibiting HIF-1α, then *LSH* as an oncogene epigenetically increases the expression level of lipid metabolic genes that inhibit ferroptosis in lung cancer (183). Reconstitution of the functional vov Hippel-Lindau (*VHL*) gene prevents ferroptosis by reverting cells back to an oxidative metabolism and increasing fatty acid degradation through β-oxidation (209). Steroyl-CoA desaturase 1 (SCD1), the rate-limiting enzyme of MUFA synthesis, is highly expressed in ovarian cancer tissues and cell lines, and overexpression of SCD1 could protect cells from ferroptosis (205).

Posttranslational modifications can also regulate ferroptosis. O-linked β-N-acetylglucosamine glycosylation of c-JUN antagonizes ferroptosis by inhibiting GSH synthesis in BEL-7402 and SMMC-7721 cells (165). Nedd4 ubiquitylates voltage-dependent anion channels 2/3 (VDAC2/3) and degrades them to suppress erastin-induced ferroptosis in melanoma cells (199).

Several genes and proteins related to ferroptosis can be utilized as prognostic markers. *G12D* is the most frequent mutation in *KRAS* (referred as KRAS^G12D^), and it is confirmed that KRAS^G12D^ could be released from PDAC cells succumbing to autophagy-dependent ferroptosis, and then KRAS^G12D^ causes macrophages to switch to an M2-like pro-tumor phenotype *via* STAT3-dependent fatty acid oxidation and it is also found that high KRAS^G12D^ expression in macrophages is correlated with poor survival in PDAC patients (173). High expression of Serine Threonine Tyrosine kinase 1 (STYK1) predicts poorer prognosis and is related to high levels of GPX4 in NSCLC (184).

### RNA-Regulated Ferroptosis

Many microRNAs (miRNAs) can protect cancer cells from ferroptosis. miRNA-522, regulated by heterogeneous nuclear ribonucleoprotein A1 (hnRNPA1), is secreted from cancer-associated fibroblasts and packed into exosomes, leading to chemo-resistance through targeting 15-LOX and decreasing ROS accumulation in gastric cancer cells (196). The inhibitory effect of miRNA-103a-3p on GLS2 is downregulated to promote ferroptosis and anti-tumorigenesis on physcion 8-O-β-glucopyranoside (PG) treatment (194). miRNA-9 can suppress GOT1 by directly binding to its 3’-untranslated region to reduce erastin- or RSL3-induced ferroptosis (197). miRNA-137 negatively regulates ferroptosis by directly targeting SLC1A5 to decrease glutamine uptake and MDA accumulation (198). Nonetheless, there are also some miRNAs that promote ferroptosis; for instance, miRNA-214-3p could promote ferroptosis by inhibiting ATF4 *in vitro* and *in vivo* (161).

A few long non-coding RNAs (lncRNAs) may also regulate ferroptosis. Erastin could upregulate the lncRNA GABPB1-AS1, which could downregulate the level of GABPB1 by blocking GABPB1 translation, leading to the downregulation of peroxiredoxin-5 and suppression of the cellular anti-oxidant capacity, and high expression of GABPB1 has been correlated with poor prognosis in HCC patients (166). The lncRNA LINC00336 is upregulated in lung cancer and functions as an inhibitor of ferroptosis in carcinogenesis by interacting with ELAV-like RNA-binding protein 1, which acts as a novel regulator of ferroptosis (185).

Several circular RNAs (circRNAs) might participate in the ferroptosis of cancer cells. Levels of circRNA TTBK2 and integrin subunit β 8 (ITGB8) are upregulated in glioma tissues and cells with downregulated miRNA-761 levels, and circRNA TTBK2 regulates cell proliferation, invasion, and ferroptosis *via* the miRNA-761/ITGB8 axis (180). circRNA 0008035, which is upregulated in gastric cancer tissues and cells, promotes the growth of gastric cancer cells and represses ferroptosis by upregulating eukaryotic initiation factor 4A1 (EIF4A1) through sponging miRNA-599 (195).

## Conclusion and Perspective

In recent years, numerous studies have been conducted to study ferroptosis in cancers, and several anti-tumor drugs (such as sorafenib, SAS, altertamin) have been approved by the FDA. However, many unsolved issues regarding ferroptosis still exist. What is the executor of ferroptosis in cancer? Which is the main pathway of lipid peroxidation in ferroptosis, the nonenzymatic pathway or enzymatic one? Does there exist another mechanism of ferroptosis resistance? What is the likelihood that the ferroptosis-related anti-tumor drugs could be used in clinical settings, such as post-operation, chemo-resistance, or radio-resistance instances? What are the potential adverse effects of ferroptosis-related anti-tumor drugs? Could the administration of ferroptosis-related anti-tumor drugs be combined with immunotherapy? The research on ferroptosis is still in its infancy, and more studies are needed to comprehensively explore the mechanism of ferroptosis in various cancers.

## Author Contributions

ZS and CS designed and conceptualized the review. All authors contributed to the article and approved the submitted version.

## Conflict of Interest

The authors declare that the research was conducted in the absence of any commercial or financial relationships that could be construed as a potential conflict of interest.
